# Straw mulching-driven microbial relay enhances soil C–N coupling and cotton yield–quality synergy

**DOI:** 10.3389/fpls.2025.1671192

**Published:** 2025-10-29

**Authors:** Zhangshu Xie, Yeling Qin, Xiaodong Xie, Jiarui Li, Lijuan Zheng, Youhong Jiang, Xiaoju Tu, Aiyu Liu, Zhonghua Zhou

**Affiliations:** ^1^ Cotton Research Institute, Agronomy College, Hunan Agricultural University/Yue Lu Shan Laboratory, Changsha, China; ^2^ Hainan Health Vocational College, Haikou, China; ^3^ Agricultural and Rural Bureau of Hengnan County, Hengyang, China; ^4^ Agricultural and Rural Bureau of Hengyang County, Hengyang, China

**Keywords:** cotton straw return, carbon–nitrogen coupling, microbial metabolic succession, yield components, fiber quality, soil ecological health

## Abstract

**Introduction:**

Straw return is a widely endorsed sustainable agronomic practice. However, a systematic understanding of its carbon–nitrogen coupling mechanisms and their consequent impacts on the soil–microbe–plant continuum across the entire cotton growth cycle is critically lacking.

**Methods:**

We conducted a field experiment with five treatments: CK (no straw return), T1 (one-third shredded straw), T2 (two-thirds shredded straw), T3 (full shredded straw), and T4 (full straw left intact as surface mulch). This design enabled us to decipher how the amount and fragmentation of straw residues synchronize the soil-microbe-plant system to enhance sustainability.

**Results:**

Our findings reveal distinct mechanistic pathways. The T3 treatment (full shredding) triggered an early-season microbial "relay," where *Gammaproteobacteria* expansion was succeeded by *Actinobacteria*, elevating soil pH from 4.82 to 5.73 and boosting alkaline-hydrolysable N by 113.01% at the flower and boll stage. This enhanced nitrate reductase activity by 74.1% and increased bolls per plant by 35.0%. In contrast, the T4 treatment (surface mulch) provided a more gradual nitrogen release (+28.4% alkaline-hydrolysable N during boll opening), which prolonged the secondary cell wall deposition phase in fibers. This strategy achieved a lint yield of 2055.63 kg ha⁻¹ (+63.8%) and a 2.6% increase in fiber strength. Furthermore, T4 fostered a "microbial sanctuary" at boll opening, evidenced by a 130.5% explosion in OTU richness and an 18.7% suppression of pathogen populations.

**Discussion:**

We demonstrate that surface mulching (T4) is the superior strategy, as it optimally balances high yield with superior fiber quality by creating a resilient and suppressive soil microbiome. This work provides a novel carbon–nitrogen synergy framework for the resource-efficient utilization of crop residues in sustainable cotton production.

## 1 Introduction

Cotton (*Gossypium hirsutum* L.), being the world’s most important natural fibre crop, must be produced stably to safeguard raw-material supplies for the textile industry and to sustain the livelihoods of growers in major production regions, particularly in developing countries. Within China, the cotton industry underpins food-and-fibre security, rural prosperity, and job creation, forming a strategic pillar of national development (Luo et al., 2014). However, the rising frequency of extreme weather linked to climate change, accelerated degradation of arable land, and increasing water scarcity have placed the conventional high-input cotton system under severe pressure; its hefty environmental costs (e.g., declining soil fertility and heightened non-point pollution risk) and inefficient resource use have become increasingly apparent (Luo et al., 2016; Li et al., 2021). As the world’s largest producer and consumer of cotton, China must reconcile the imperative of secure fibre supply with the need to ease environmental pressure. For example, in Hunan Province—a traditional cotton belt of the Yangtze River basin—short-season cotton systems are long constrained by a yield plateau, fluctuations in fibre quality, and low efficiencies in water and nutrient use (Lu et al., 2022). Consequently, developing and scaling up resource-efficient, environmentally benign and profitability-enhancing cotton technologies has become an urgent requirement for the sector’s sustainable development.

Transitioning cotton cultivation to sustainable models bears far-reaching implications for advancing the country’s green-development agenda, safeguarding fibre security, and mitigating intensifying environmental challenges (Dai et al., 2017). Amongst sustainable agronomic practices, straw return to the field has garnered particular attention owing to its pronounced ecological benefits and value in resource cycling (Tang et al., 2023). By scientifically reincorporating crop residues into the soil, this practice can, in theory, elevate soil organic matter, improve physical structure—such as increasing porosity and aggregate stability—enhance soil water- and nutrient-holding capacity, facilitate the cycling and retention of nutrients (especially nitrogen), and markedly activate and regulate the composition and function of soil microbial communities, thereby building a more productive and resilient agroecosystem (Ma et al., 2019; Yang et al., 2024). Healthy soil is regarded as the essential foundation for simultaneously achieving high cotton yields, superior fibre quality, and efficient resource use.

Previous studies in various cropping systems, such as rice (Jin et al., 2020), wheat (Huang et al., 2019), and maize (Yang et al., 2020), have demonstrated that returning straw to the field improves soil physicochemical properties. These improvements include increases in organic carbon and available nutrients, as well as enhancements in certain biological indicators. For cotton, research has similarly reported that straw incorporation positively impacts specific soil parameters in the short term, such as C and N contents, as well as particular microbial groups (Li and Zhang, 2016). It also affects certain growth-stage indicators of the crop.

However, substantial gaps remain. Most investigations focus on simple observations of straw return effects on one or a few soil indicators. These studies lack in-depth exploration of how residue incorporation drives the integrated evolution of the cotton-field soil ecosystem. This includes changes in physical structure, chemical properties, and biotic communities, along with their interactions (Pu et al., 2016). More critically, research on the mechanistic pathways by which straw-induced soil ecological changes are quantitatively transmitted throughout the cotton life cycle is still limited. These pathways alter key agronomic traits such as root development dynamics, canopy-building efficiency, photosynthetic performance, and reproductive differentiation. Ultimately, they govern yield components, including boll number, single-boll weight, lint percentage, and core fibre-quality metrics like length, strength, uniformity, and micronaire (Hu et al., 2021).

Moreover, studies on the short-season cotton system in Hunan Province are particularly scarce. This system has a compressed growth period, which imposes stringent spatiotemporal demands on water and fertiliser resources. The influence of straw return on soil moisture–nutrient dynamics and nutrient provisioning under these conditions is unclear. It is also uncertain how these changes align with the crop’s rapid growth requirements. Additionally, there is a lack of evidence to guide the differential effects and optimisation of various residue-return practices, such as degrees of shredding and incorporation rates, within the short-season cultivation system (Yang et al., 2013). At the same time, our understanding of how straw return shapes the soil microecology, including microbial diversity and functional guild structures, remains limited. Furthermore, the potential effects of these shifts on cotton growth and development are still not well understood (Liu et al., 2012).

Accordingly, this study centres on a representative short-season cotton region in Hunan Province, where a field experiment with multiple residue-return gradients and two incorporation modes (shredded vs. surface mulch) was established to systematically construct and decipher the intrinsic links along the full chain of “soil ecological evolution → cotton physiological responses → final productive performance.” The work delineates how straw return drives systemic soil transformations under the short-season regime and elucidates the key mechanisms that ultimately regulate cotton yield and fibre quality. Its principal innovation lies in the deployment of integrative, multidimensional monitoring to refine our understanding of how straw return under short-season cotton shapes overall soil ecosystem functionality and transmits its effects to crop productivity and quality.

The present investigation seeks to tackle the following critical issues: 1) How do varying straw-return regimes—in terms of shredding intensity and incorporation rate—modify core soil attributes, as revealed by changes in chemical factors (e.g., pH, organic matter, nutrient-buffering capacity) and biological metrics (e.g., microbial biomass, community structure)? 2) How are the soil ecological shifts triggered by straw incorporation transmitted to cotton, influencing key agronomic traits such as plant height, fruiting-branch number, and height of the first fruiting branch? 3) How do soil amelioration and improved plant traits translate into gains in yield components (boll number, single-boll weight, lint percentage) and fibre-quality metrics (length, strength, uniformity, micronaire)? 4) Within the short-season cultivation system, what combination of shredding intensity and return rate constitutes the optimal straw-return strategy, and what regulatory mechanisms underpin its efficacy?

Accordingly, a field experiment was established in Yanxi Town, Liuyang City, Hunan Province, comprising five straw-return treatments: no return (control, CK), one-third shredded straw return (T1), two-thirds shredded straw return (T2), full-rate shredded straw return (T3), and full-rate unshredded straw left as surface cover (T4). By monitoring soil ecological parameters, cotton agronomic traits, and both yield and fibre quality—and subjecting the data to statistical analyses—we elucidated the intrinsic links between straw incorporation and changes in cotton production performance, thereby providing a scientific basis for developing an efficient and sustainable straw-return technology system for short-season cotton in the Yangtze River basin.

## 2 Materials and methods

### 2.1 Experimental materials

The experimental cotton cultivar was JX0010, an early-maturing, transgenic insect-resistant line (growth period: 105 days) developed by the Cotton Research Institute of Hunan Agricultural University. Before planting, the seeds were delinted using concentrated H_2_SO_4_, sorted for size uniformity, and sun-dried, and only evenly sized kernels were retained for sowing.

### 2.2 Overview of the test site

The trial took place at the Liuyang Internship and Experimental Farm of Hunan Agricultural University (28°18′19″N, 113°49′26″E). The area features a warm, humid subtropical monsoon climate, characterised by ample light and heat and plenty of precipitation, providing optimal conditions for cotton growth.

### 2.3 Experimental design

On 1 December 2022, the standing cotton field was partitioned into 15 experimental subplots, and the entire aboveground cotton residue was gathered from each. Each plot covered 20 m² (5 m × 4 m), was planted at 60,000 plants ha^−1^, and thus contained residues from 120 plants.

The collected straw was sun-dried and then comminuted with a model 1010 crusher (Zhengzhou Guruite Machinery Co., Henan, China) to fragments <30 mm. Five treatments were imposed—no straw return (CK), one-third shredded straw return (T1), two-thirds shredded straw return (T2), full-rate shredded straw return (T3), and full-rate unshredded surface return (T4)—each with three replicates, totalling 15 plots. On 15 December 2022, the plots were ploughed to a depth of 25 cm, and the designated quantities of straw were incorporated. Sowing took place on 20 May 2023 at 60,000 plants ha^−1^, and subsequent crop management adhered to standard short-season direct-sowing practices (DB43/T 2379-2022; Hunan Provincial Bureau of Technology and Quality Supervision, 2022).

### 2.4 Measurement indicators and methods

#### 2.4.1 Physiological indicators

At four growth stages (seedling, bud, flower and boll, and boll opening), five morphologically similar cotton plants were tagged in each plot. The fourth leaf from the apex on the main stem was excised, immediately flash-frozen in liquid nitrogen, and transported to the laboratory. Nitrate-reductase and peroxidase activities were then quantified using commercial assay kits (Sangon Biotech Co., Shanghai, China) according to the manufacturer’s instructions.

#### 2.4.2 Agronomic traits

At the seedling, bud, flower–boll, and boll-opening stages, 10 morphologically uniform plants were tagged in each plot.

Plant height was determined with a tape measure as the vertical distance from the soil surface to the shoot apex.

Fruiting-branch number was counted on the main stem at the bud, flower–boll, and boll-opening stages.

Height of the first fruiting branch was measured vertically from the cotyledonary node to the insertion point of the first fruiting branch.

#### 2.4.3 Assessment of soil physicochemical characteristics and microbial community composition

Soil samples were collected at the seedling, bud, flower–boll, and boll-opening stages. A soil auger with a 4-cm inner diameter and 60-cm length was used to collect cores from five points per plot, with three replicates that were subsequently composited. Each composite was sealed in zip-lock bags and transported on ice to the laboratory; one portion was air-dried, ground, and passed through 20- and 100-mesh sieves for determination of pH, organic matter, total N, and alkaline-hydrolysable N, whereas the other portion was stored at −80°C for microbial community analyses.

Soil pH was measured with a pH meter (model TP140, Beijing Time New Vision Measurement & Control Co., Beijing, China).

Soil organic matter was quantified by the potassium-permanganate oxidation method following de Sousa et al (de Sousa et al., 2025).

Total N and alkaline-hydrolysable N was determined according to the methods of Drescher et al. (2020) and Greenfield (2001).

Bacterial community (class level) was characterised by sequencing of the 16S V4 region using an Illumina PE250 platform. DNA was extracted from soil samples (0–20 cm) using the E.Z.N.A. Soil DNA Kit (Omega Bio-tek, Inc., USA) according to the manufacturer’s protocol. The bacterial 16S rRNA gene V3–V4 hypervariable region was amplified using the universal primers 338F (5′-ACTCCTACGGGAGGCAGCAG-3′) and 806R (5′-GGACTACNNGGGTATCTAAT-3′). PCR amplification was performed using the following conditions: an initial denaturation at 95 °C for 5 min, followed by 28 cycles of 95 °C for 45 s, 55 °C for 50 s, and 72 °C for 45 s, with a final extension at 72 °C for 10 min. PCR products were purified and sequenced on an Illumina platform, as described by Xie et al (Xie et al., 2025).

#### 2.4.4 Yield survey

Within each test plot, representative samples were randomly selected under a uniform-distribution scheme. At the boll-opening stage, 50 open bolls were harvested per plot, sun-dried, and weighed for seed-cotton mass; lint mass was recorded after ginning.

Boll number per plant: total number of bolls on an individual plant.

Single-boll weight: mean dry weight of the 50 sampled bolls.

Lint percentage: lint mass as a percentage of seed-cotton mass.


$$\begin{array}{l} Seed-Cotton\;yield(kg\;ha^{-1})=plant\;density(plants\;ha^{-1})\\ \times bolls\;plant^{-1}\times \text{single}-boll\;weight(g)/1,000\\ \times correction\;factors\;0.85. \end{array}$$

$$\begin{array}{c} Lint\;yield(kg\;ha^{-1})=seed-cotton\;yield(kg\;ha^{-1})\\ \times lint\;percentage(\%). \end{array}$$

#### 2.4.5 Fibre quality testing

From each plot, 50 open bolls were picked from the mid-lower canopy, sun-dried, and ginned to obtain lint samples. A 30-g subsample was sent to the Institute of Cotton Research, Chinese Academy of Agricultural Sciences (Henan, China), where fibre quality traits—including uniformity index, fibre strength, average length of upper half, micronaire, and elongation—were measured with a high-capacity HVI 1000 rapid fibre tester.

### 2.5 Data processing and analysis

Data were collated in Microsoft Excel 2010, and one-way analysis of variance (ANOVA) was conducted using SPSS Statistics 25.0 (IBM Corporation, USA). Duncan’s test was used for significance analysis, and plots were created using Origin 2022. Bacterial 16S sequences were clustered into OTUs following the method of Rognes et al. (2016) and Edgar (2013), and subsequent calculations were conducted with QIIME v1.8.0 (Caporaso et al., 2010).

## 3 Results and analysis

### 3.1 Changes in nitrate reductase activity under different treatments

**Figure 1:**
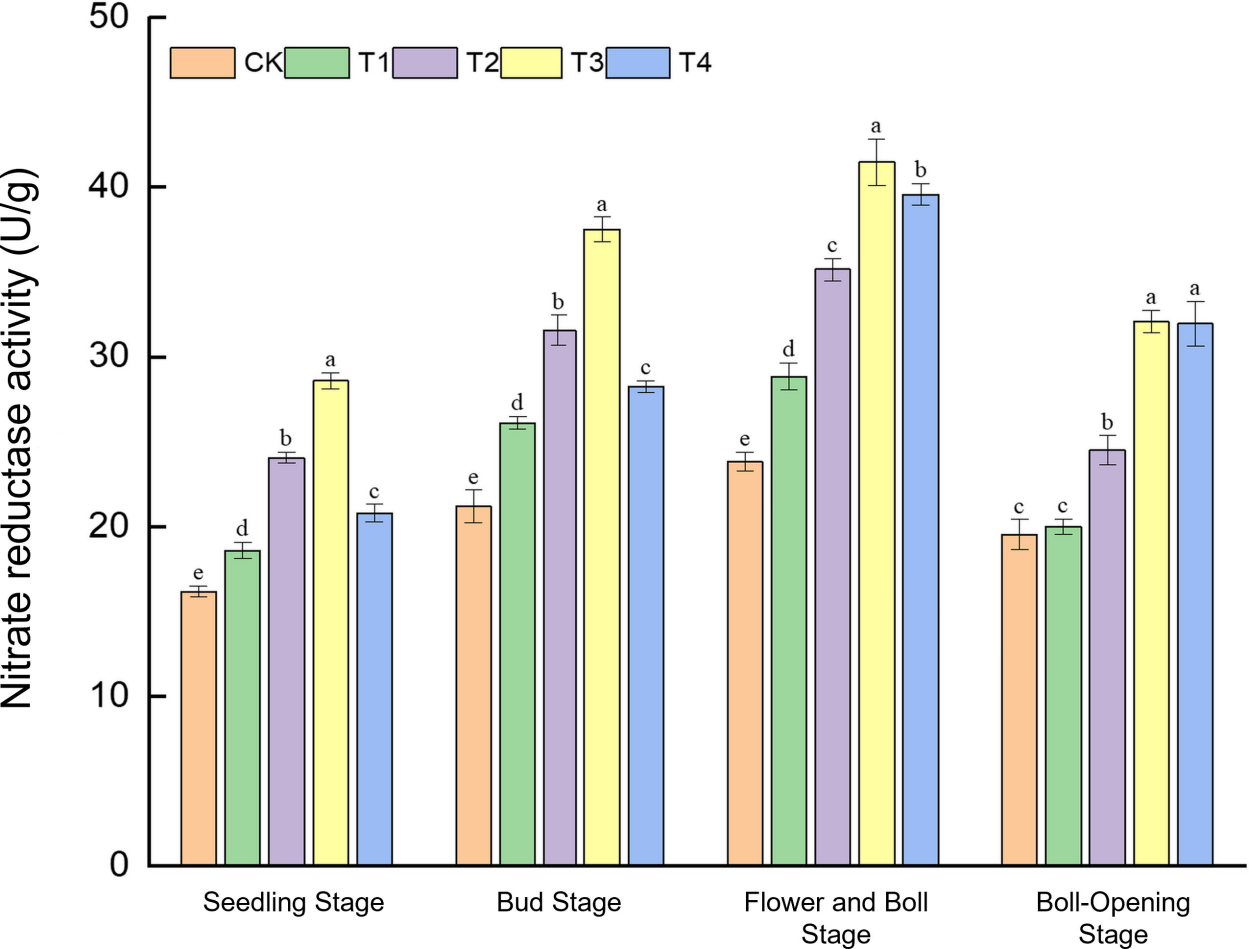
Effects of different treatments on nitrate reductase activity (U g^−1^) in cotton leaves. Different lowercase letters indicate significant differences in mean values between different treatments during the same growth stage, as determined by Duncan’s one-way ANOVA (*p* < 0.05).

The temporal dynamics of nitrate reductase (NR) activity were significantly influenced by both the rate of straw incorporation and the physical form of the straw (shredded vs. unshredded), with a significant interaction between these two factors observed across the growth cycle (**Figure 1**).

Regarding the main effect of incorporation rate, NR activity exhibited a strong positive response to increasing amounts of straw from the seedling to the flower–boll stage. Across these stages, the general trend followed CK < T1 < T2 < T3, indicating that a higher carbon substrate from straw enhanced the plant’s nitrogen metabolic capacity.

Conversely, the main effect of the straw form became particularly pronounced at the boll-opening stage. At this late developmental phase, treatments involving shredded straw (T3 and T4) sustained significantly higher NR activity compared to their unshredded counterparts at equivalent incorporation rates.

The significant interaction between rate and form was most evident when comparing the high-rate treatments (T3 vs. T4). While the full-rate shredded treatment (T3) consistently provided the greatest advantage from seedling through flower–boll stages, its superiority diminished by boll opening, where the half-rate shredded treatment (T4) achieved a comparable level of NR activity. This suggests that shredding can compensate for a lower incorporation rate in maintaining nitrogen metabolism during the late reproductive phase.

At the seedling stage, NR activity under T3 (28.59 U g^−1^) was 76.8% higher than the control (CK, 16.17 U g^−1^). During the bud stage, T3 reached 37.52 U g^−1^, a 48.9% increase over CK (25.20 U g^−1^). The peak NR activity was observed at the flower–boll stage, where T3 attained 41.48 U g^−1^—74.1% higher than CK (23.82 U g^−1^), thereby robustly meeting the high nitrogen demand of boll development. Finally, at boll opening, the activities in T3 (32.08 U g^−1^) and T4 (31.98 U g^−1^) were statistically comparable, yet both were approximately 64.2% above CK (19.54 U g^−1^). The consistent superiority of the T3 treatment is attributed to the rapid nitrogen release from the high rate of shredded residues, which optimally synchronised nitrogen availability with crop demand.

### 3.2 Changes in peroxidase activity under different treatments

**Figure 2:**
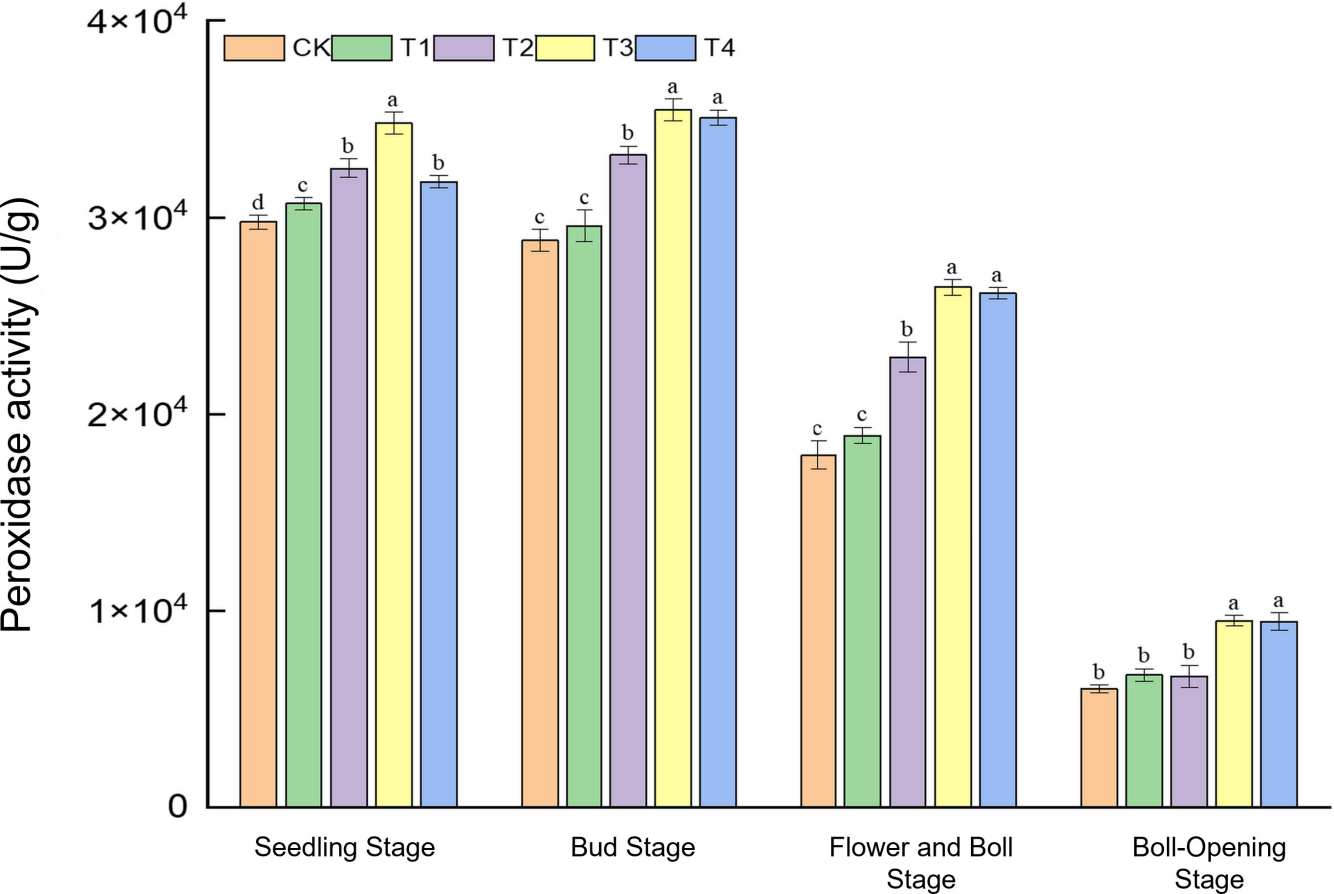
Effects of different treatments on peroxidase activity (U g^−1^) in cotton leaves. Different lowercase letters indicate significant differences in mean values between different treatments during the same growth stage, as determined by Duncan’s one-way ANOVA (*p* < 0.05).

Peroxidase (POD), a key enzyme for reactive-oxygen scavenging, serves as an indicator of the crop’s antioxidative capacity. As shown in **Figure 2**, straw incorporation markedly modulated POD activity at all growth stages, with full-rate shredded return (T3) and surface mulching (T4) exhibiting the strongest effects during mid-to-late development. At the seedling stage, POD activity ranged from 2.97 × 10^4^ to 3.48 × 10^4^ U g^−1^; T3 peaked, showing a 16.9% rise over CK and exceeding T1, T2, and T4 by 3.1%, 9.1%, and 6.9%, respectively. During the bud stage, activities (2.88 × 10^4^–3.55 × 10^4^ U g^−1^) under T3 and T4 were 23.1% and 21.6% higher than CK. At the flower–boll stage, POD reached its maximum (1.79 × 10^4^–2.65 × 10^4^ U g^−1^) under T3 and T4, 47.7% and 46.1% above CK. Even as activities declined to 0.60 × 10^4^–0.95 × 10^4^ U g^−1^ at boll opening, T3 and T4 maintained significant advantages, whereas CK, T1, and T2 did not differ. Overall, only high-dose shredded return (T3) boosted POD at the seedling stage; thereafter, T3 and T4 acted synergistically, with the largest (>46%) enhancement at the flower–boll stage. Continuous residue decomposition evidently improved the rhizosphere environment and reinforced the cotton antioxidative system. Sustained POD upregulation under full-rate return (T3/T4) provides physiological protection against oxidative stress during reproduction.

In summary, only the high-dose shredded straw return (T3) markedly enhanced POD activity at the seedling stage. After the bud stage, T3 and T4 acted synergistically, with the largest increase occurring at the flower–boll stage (>46%), owing to continuous straw decomposition that improved the rhizosphere environment and strengthened the cotton antioxidative system. Full-rate straw return (T3/T4) thus consistently reinforced enzyme activity during the mid-to-late season, delivering physiological protection against oxidative stress in the reproductive phase.

### 3.3 Changes in agronomic traits under different treatments

Straw incorporation markedly optimised key agronomic traits of cotton, with the full-rate shredded treatment (T3) exhibiting the most pronounced effects (**Table 1**). Plant height remained superior under T3: at the seedling stage, it reached 37.73 cm, 26.3% higher than the control (CK, 29.87 cm); at the bud stage, it was 52.67 cm, 15.8% above CK (45.47 cm); and at the flower–boll and boll-opening stages, it rose to 99.33 cm and 130.74 cm, representing increases of 11.4% and 10.5% over CK, respectively, and significantly exceeding all the other treatments (*p* < 0.05). Fruiting-branch number was likewise dominated by T3: at the bud stage, it reached 8.64, 56.2% greater than CK (5.53); at the flower–boll and boll-opening stages, it rose to 14.11 and 13.40, corresponding to gains of 37.4% and 36.6%, respectively. The height of the first fruiting branch was the highest under T3 at boll opening (26.98 cm), 38.0% higher than CK (19.55 cm).

**Table 1:** Effects of different treatments on cotton agronomic traits.

Items	Treatment	Seedling stage	Squaring stage	Flower and boll stage	Boll-opening stage
Plant height (cm)	CK	29.87 ± 3.76c	45.47 ± 3.13b	89.13 ± 2.27c	118.27 ± 3.51c
	T1	29.31 ± 2.52c	48.13 ± 2.91ab	93.27 ± 2.99bc	125.26 ± 3.32b
	T2	33.33 ± 3.31b	49.33 ± 3.06ab	95.47 ± 2.72ab	125.27 ± 2.62b
	T3	37.73 ± 2.11a	52.67 ± 2.61a	99.33 ± 2.61a	130.74 ± 1.88a
	T4	34.46 ± 2.83b	51.11 ± 2.31ab	96.35 ± 2.82ab	129.86 ± 2.47a
Number of fruit branches	CK	–	5.53 ± 0.64c	10.27 ± 0.61c	9.81 ± 1.06c
	T1	–	6.47 ± 0.91bc	11.73 ± 1.36bc	10.81 ± 0.61bc
	T2	–	6.93 ± 0.87b	12.40 ± 0.51b	12.27 ± 0.61ab
	T3	–	8.64 ± 1.02a	14.11 ± 0.56a	13.40 ± 1.25a
	T4	–	7.36 ± 0.71b	12.39 ± 1.05b	12.73 ± 1.03a
Height of first fruit branch (cm)	CK	–	–	–	19.55 ± 2.48b
	T1	–	–	–	23.63 ± 1.43ab
	T2	–	–	–	24.25 ± 2.71ab
	T3	–	–	–	26.98 ± 3.35a
	T4	–	–	–	24.87 ± 3.39ab

Different lowercase letters indicate significant differences in mean values between different treatments during the same growth stage, as determined by Duncan’s one-way ANOVA (*p* < 0.05).

### 3.4 Changes in soil physicochemical properties under different treatments

#### 3.4.1 Changes in soil pH

**Figure 3:**
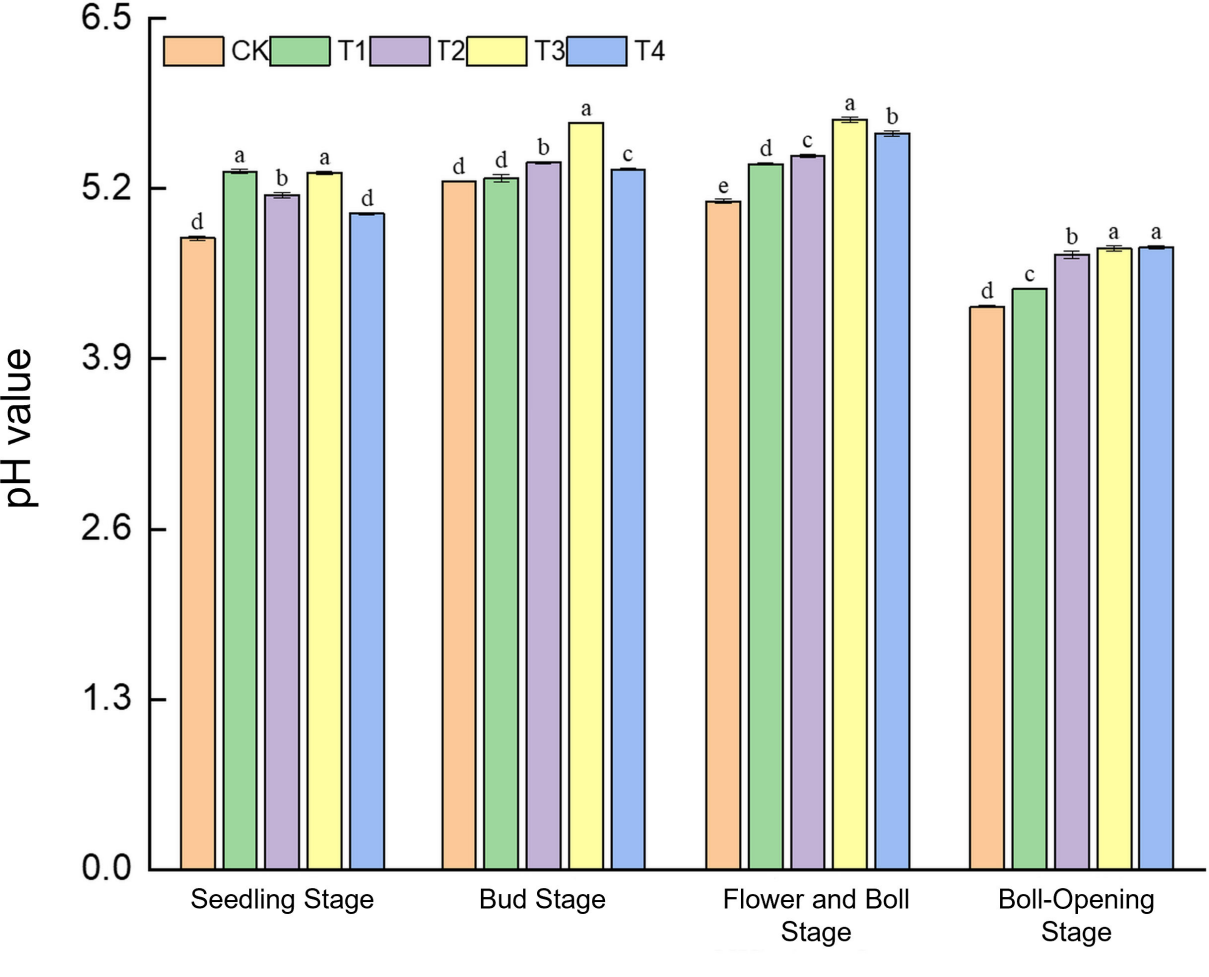
Effects of different treatments on soil pH in cotton fields. Different lowercase letters indicate significant differences in mean values between different treatments during the same growth stage, as determined by Duncan’s one-way ANOVA (*p* < 0.05).

Straw incorporation markedly alleviated soil acidification in cotton fields, with the full-rate shredded treatment (T3) showing the most consistent effect (**Figure 3**). At the seedling stage, soil pH (4.82–5.33) under T1 and T3 was 1.0% higher than CK, whereas CK and T4 did not differ. During the bud stage, T3 reached a pH peak of 5.71, an 8.6% increase over CK (5.26). By the flower–boll stage, pH values across treatments (5.11–5.73) were all significantly higher than CK, with T2 exhibiting the greatest rise (12.2%) and T3 the second-largest (10.0%). At boll opening, pH under T3 (4.75) and T4 (4.74) surpassed CK (4.30) by 10.5%. These results indicate that from the bud to boll-opening stages, straw return continuously elevates soil pH, and T3 maintains significant pH enhancement throughout the growth period, underscoring its steady regulation of soil acid–base balance.

#### 3.4.2 Changes in soil organic matter content

**Figure 4:**
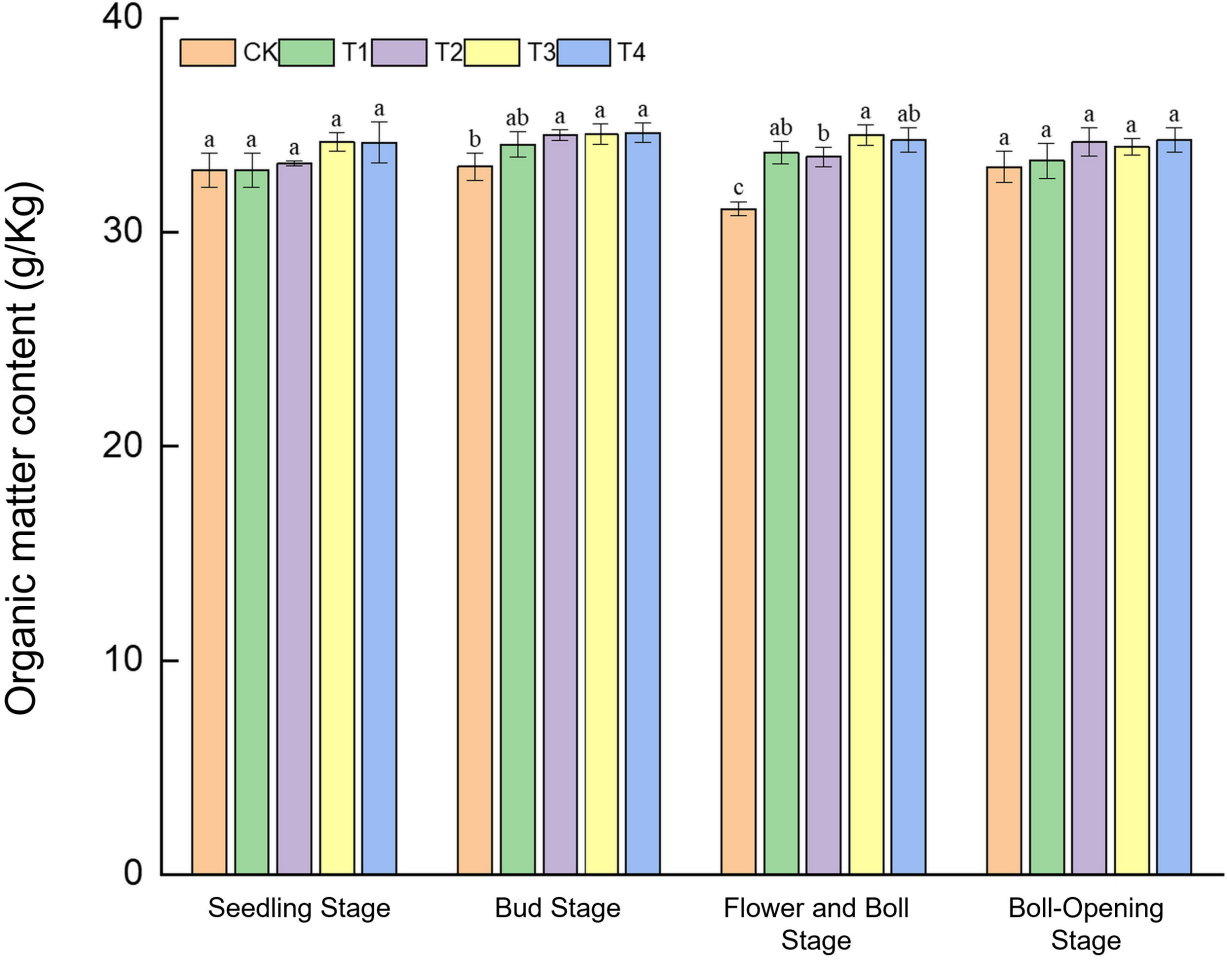
Effects of different treatments on soil organic-matter content in cotton fields. Different lowercase letters indicate significant differences in mean values between different treatments during the same growth stage, as determined by Duncan’s one-way ANOVA (*p* < 0.05).

Straw incorporation enhanced soil organic matter in a growth-stage-dependent manner (**Figure 4**). At the seedling stage, organic-matter contents amongst treatments (32.9–34.21 g kg^−1^) did not differ significantly, although T3 was marginally higher (34.21 g kg^−1^). During the bud stage, contents under T2–T4 (34.47–34.65 g kg^−1^) exceeded CK (33.08 g kg^−1^) by 4.2%–4.7%. The greatest improvements occurred at the flower–boll stage: T3 peaked at 34.55 g kg^−1^, 7.91% above CK (31.09 g kg^−1^), while T1, T2, and T4 were likewise 7.8%–10.3% higher than CK. By boll opening, differences disappeared, with values converging to 33.05–34.30 g kg^−1^. These results indicate a phased release pattern of straw decomposition—initial effects emerging at the bud stage and maximum response at the flower–boll stage—confirming the carbon-supply advantage of full-rate shredded return during the peak nutrient-demand period.

#### 3.4.3 Changes in soil total nitrogen content

**Figure 5:**
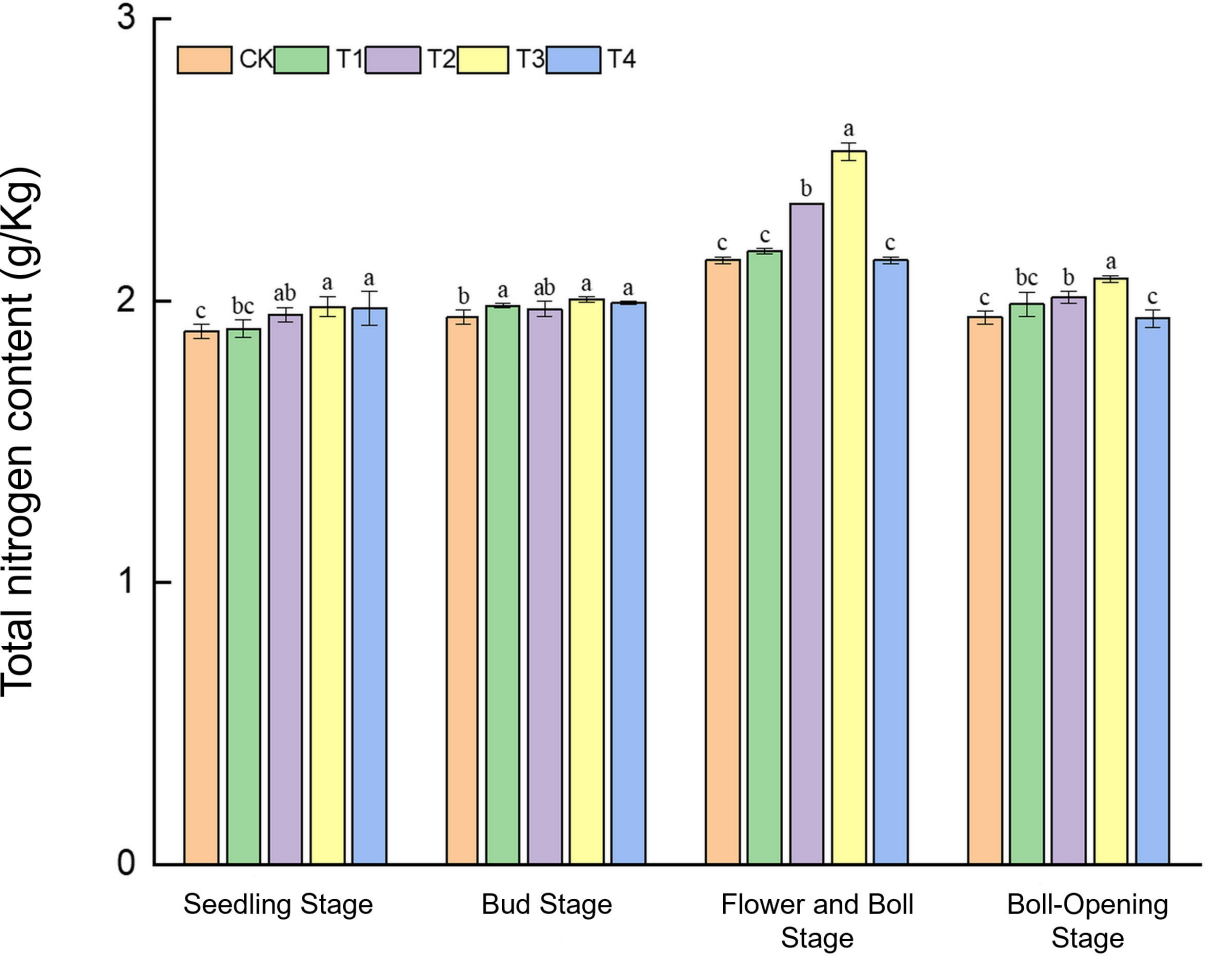
Effects of different treatments on total nitrogen content in cotton-field soils. Different lowercase letters indicate significant differences in mean values between different treatments during the same growth stage, as determined by Duncan’s one-way ANOVA (*p* < 0.05).

Straw incorporation markedly increased the soil nitrogen pool in cotton fields, with the full-rate shredded treatment (T3) showing pronounced advantages during the mid-to-late growth stages (**Figure 5**). During the seedling stage, total N (1.89–1.98 g kg^−1^) was the highest under T3 and T4, exceeding CK by 4.8% and 4.2%, respectively. At the bud stage, T3 reached 2.01 g kg^−1^, 3.6% higher than CK (1.94 g kg^−1^). In the flower–boll stage, T3 peaked at 2.53 g kg^−1^, a substantial 17.7% increase over CK (2.15 g kg^−1^), whereas T1 and T4 did not differ from CK. At boll opening, T3 (2.07 g kg^−1^) still exceeded CK (1.94 g kg^−1^) by 6.7%. These results indicate that the rapid mineralisation of shredded straw in T3 enabled efficient N release during the critical nitrogen-demand period (flower–boll stage), whereas the surface-mulched treatment (T4) was effective only in early growth.

#### 3.4.4 Changes in soil alkaline-hydrolysable nitrogen content

**Figure 6:**
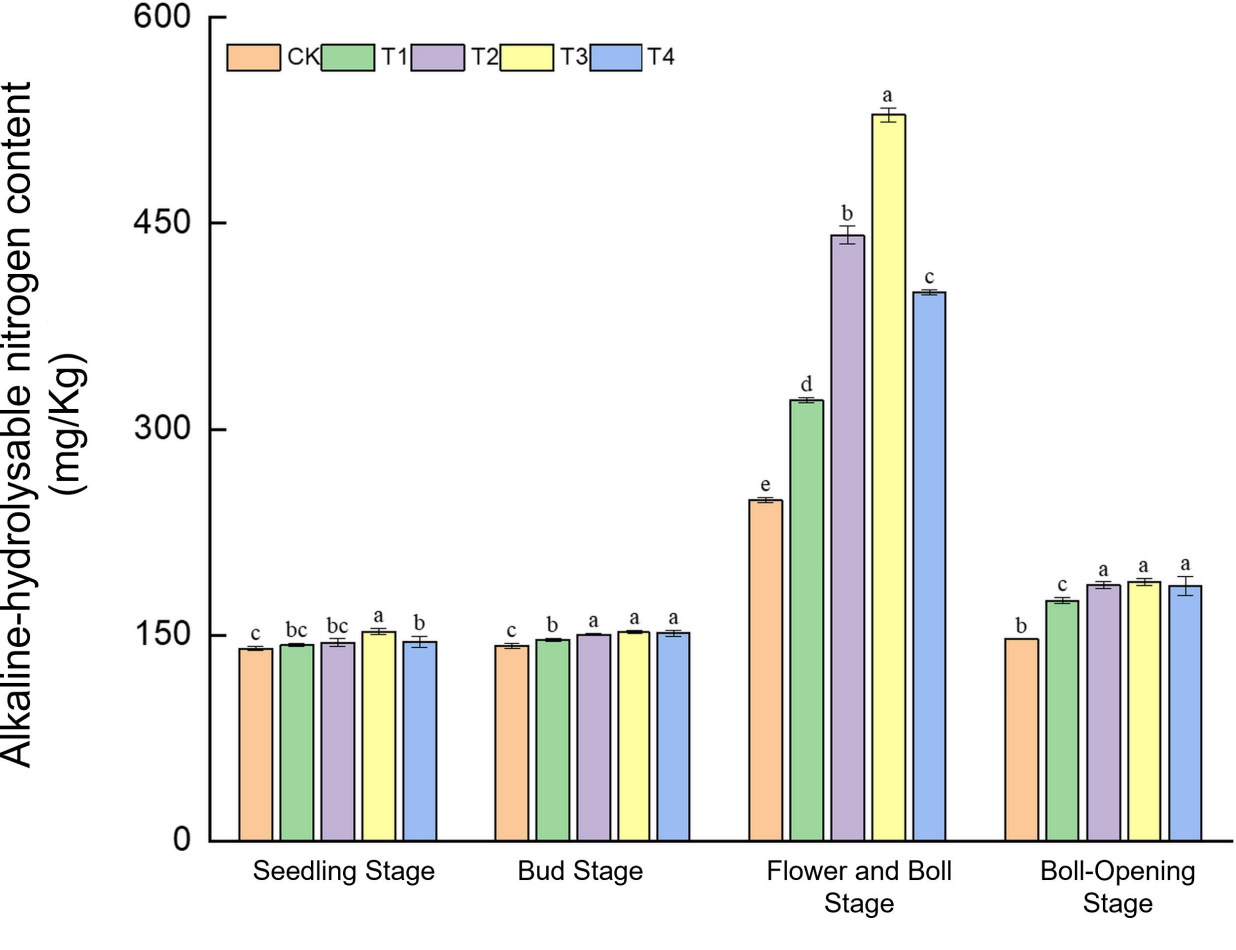
Effects of different treatments on alkaline-hydrolysable nitrogen content in cotton-field soils. Different lowercase letters indicate significant differences in mean values between different treatments during the same growth stage, as determined by Duncan’s one-way ANOVA (*p* < 0.05).

Straw incorporation markedly enhanced the supply of alkaline-hydrolysable nitrogen, with effects varying dynamically across growth stages (**Figure 6**). In the seedling stage, T3 recorded the highest level (152.76 mg kg^−1^), 8.8% above CK. During the bud stage, T2–T4 (149.44–152.53 mg kg^−1^) exceeded CK (142.44 mg kg^−1^) by 4.9%–7.1%. A dramatic response occurred at the flower–boll stage: T3 peaked at 529.15 mg kg^−1^, surging 113.0% over CK (248.40 mg kg^−1^). At boll opening, T2–T4 (186.78–188.78 mg kg^−1^) surpassed CK (147.02 mg kg^−1^) by 27.0%–28.4%. These findings show that full-rate shredded return (T3) delivered an explosive release of readily available nitrogen (+113%) during the critical flower–boll stage, whereas surface mulching (T4) worked synergistically with shredded treatments (T2/T3) at the bud and boll-opening stages.

### 3.5 Changes in the soil microbial community

#### 3.5.1 Alpha diversity indices

Straw incorporation exerted pronounced growth-stage specificity in shaping the soil microbial community (**Table 2**). From the seedling to the flower–boll stages, most treatments did not differ significantly in Shannon (9.59–10.95) or Simpson (0.99–1.00) indices. By contrast, the Chao1 richness index continued to respond: at the bud stage, T3 (8,406.90) was 6.7% higher than CK (7,877.75), whereas at the flower–boll stage, T2 (6,183.34) was 13.3% lower than CK (7,135.25). A fundamental shift occurred at boll opening: Shannon values for T3 and T4 (10.48, 10.69) exceeded CK (9.62) by 8.9% and 11.1%, respectively; the Simpson index stabilised at 1.00 for both (significantly above CK’s 0.99); and Chao1 richness in T3 (7,302.96) and T4 (7,198.48) rocketed by 34.4% and 36.3% over CK (5,356.68). Collectively, these results indicate that straw return mainly increased microbial richness in early growth (Chao1 + 6.7%), whereas by boll opening, it simultaneously enhanced diversity (Shannon + 11.1%) and richness (Chao1 + 36.3%); the full-rate treatments (T3/T4) rebuilt the microbial community through continuous carbon inputs.

**Table 2:** Soil microbial diversity and richness in cotton fields under different treatments.

Items	Treatment	Seedling stage	Bud stage	Flower and boll stage	Boll-opening stage
Shannon index	CK	10.29 ± 0.76a	10.73 ± 0.06a	10.58 ± 0.08a	9.62 ± 0.38c
	T1	10.87 ± 0.07a	10.50 ± 0.11b	10.69 ± 0.06a	10.13 ± 0.27b
	T2	10.91 ± 0.06a	10.70 ± 0.02a	9.59 ± 0.27b	10.19 ± 0.17b
	T3	10.65 ± 0.26a	10.81 ± 0.08a	10.40 ± 0.16a	10.48 ± 0.07ab
	T4	10.95 ± 0.11a	10.74 ± 0.06a	10.48 ± 0.45a	10.69 ± 0.06a
Simpson index	CK	0.99 ± 0.17b	1.00 ± 0.00a	1.00 ± 0.00a	0.99 ± 0.01b
	T1	1.00 ± 0.00a	0.99 ± 0.01b	1.00 ± 0.00a	0.99 ± 0.01b
	T2	1.00 ± 0.00a	1.00 ± 0.00a	0.99 ± 0.01b	1.00 ± 0.00a
	T3	1.00 ± 0.00a	1.00 ± 0.00a	1.00 ± 0.00a	1.00 ± 0.00a
	T4	1.00 ± 0.00a	1.00 ± 0.00a	1.00 ± 0.00a	1.00 ± 0.00a
Chao1 index	CK	7,388.11 ± 600.52b	7,877.75 ± 243.74b	7,135.25 ± 70.14a	5,356.68 ± 464.49c
	T1	8,309.38 ± 125.98a	8,195.79 ± 152.99ab	7,424.91 ± 193.87a	6,667.70 ± 208.40b
	T2	8,484.61 ± 178.37a	8,337.27 ± 284.00a	6,183.34 ± 107.25b	6,694.42 ± 163.65b
	T3	7,872.53 ± 671.81ab	8,406.90 ± 179.89a	7,009.18 ± 406.87a	7,302.96 ± 164.21a
	T4	8,257.90 ± 238.19ab	8,189.78 ± 99.43ab	7,032.72 ± 529.16a	7,198.48 ± 85.57a

Different lowercase letters indicate significant differences in mean values between different treatments during the same growth stage, as determined by Duncan’s one-way ANOVA (*p* < 0.05).

#### 3.5.2 Composition of soil microorganisms at the class level

**Figure 7:**
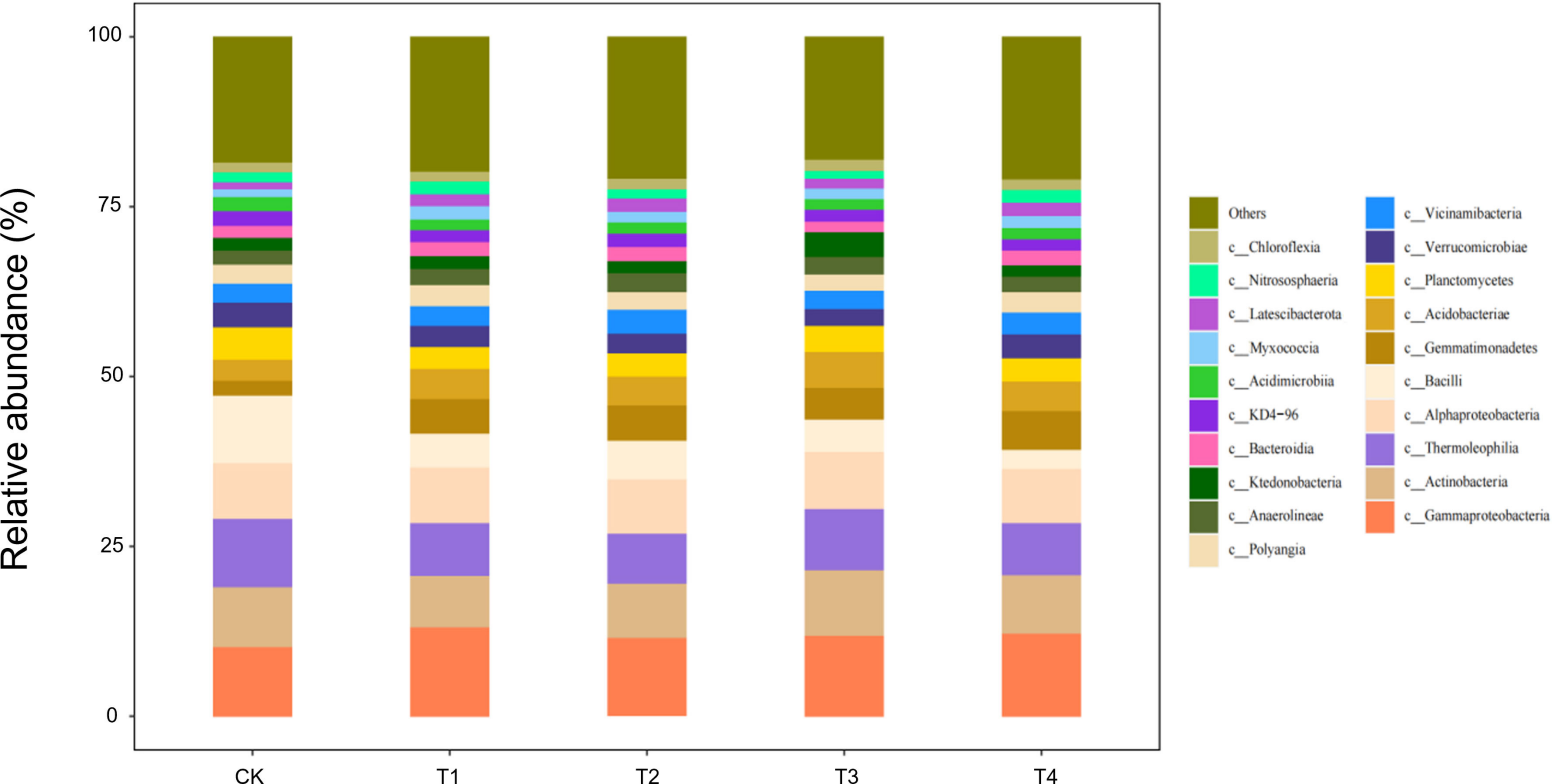
Class-level soil microbial composition at the seedling stage is altered by straw incorporation.

At the class level, 20 bacterial classes with relative abundances >1% were detected in seedling-stage soils, of which *Gammaproteobacteria*, *Actinobacteria*, and *Thermoleophilia* constituted the core community, together accounting for >65% of the reads (**Figure 7**). Straw return markedly reshaped this composition: full-rate shredded (T3) and surface-mulched (T4) treatments increased *Gammaproteobacteria* by 12%–15% and *Actinobacteria* by 8%–10%, while sharply suppressing oligotrophic groups such as *Acidimicrobiia* by 20%–30% relative to CK. This directional succession reflects a concerted expansion of carbon-cycling guilds—*Gammaproteobacteria* as primary decomposers of organic matter and *Actinobacteria* with lignocellulose-degrading capacity—jointly responding to the eutrophic microenvironment rebuilt by straw inputs.

**Figure 8:**
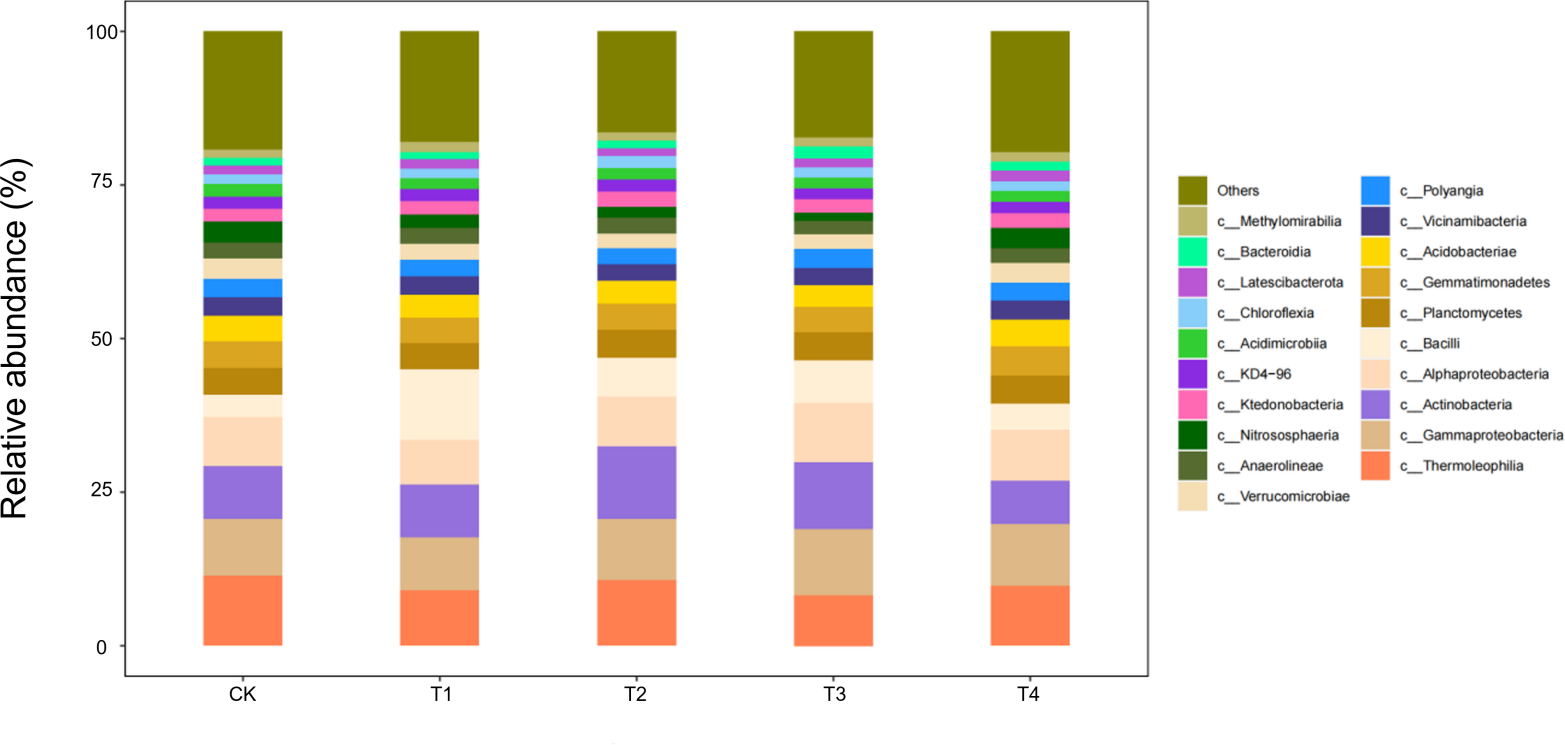
Class-level soil microbial composition at the bud stage is altered by straw incorporation.

During the bud stage, the bacterial community displayed a pronounced straw-dose effect: as the return rate increased from CK to T4, the relative abundance of *Gammaproteobacteria*—one of the core classes—rose by 35%–40%, whereas *Thermoleophilia* and *Actinobacteria* declined by 18%–22% and 12%–15%, respectively (**Figure 8**). This structural reconfiguration indicates that low straw inputs (T1/T2) help maintain the thermophilic, oligotrophic *Thermoleophilia*, whereas full returns (T3/T4) shift the community towards a *Gammaproteobacteria*-dominated, eutrophic assemblage. The latter class accelerates the breakdown of water-soluble carbon in straw by upregulating carbohydrate-metabolic pathways, thereby supplying readily available carbon for the flower-transitioning cotton plants.

**Figure 9:**
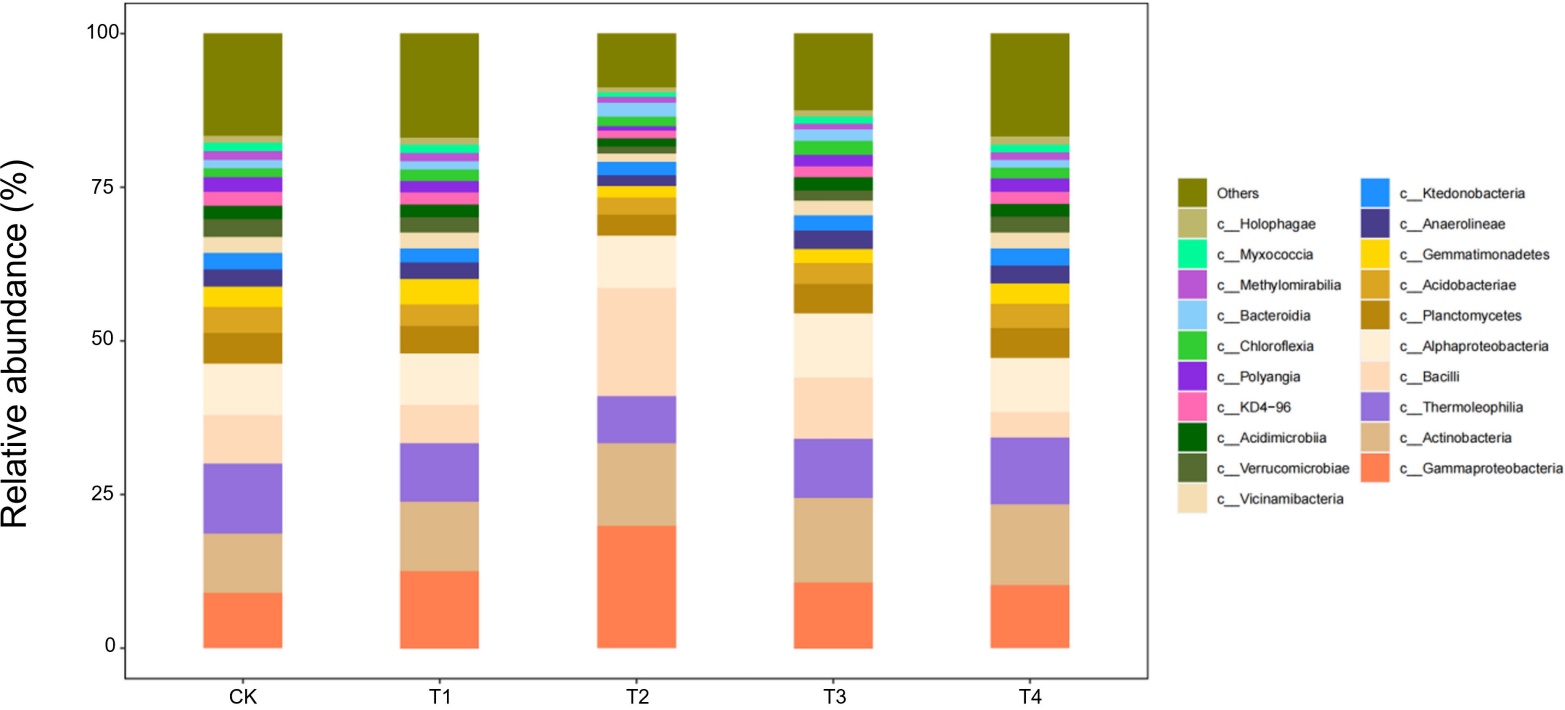
Class-level soil microbial composition at the flower–boll stage is altered by straw incorporation.

During the flower–boll stage, the soil bacterial community responded non-linearly to straw-return rates (**Figure 9**). The relative abundances of three core classes—*Gammaproteobacteria*, *Actinobacteria*, and *Bacilli*—followed a unimodal pattern, peaking under T2 (two-thirds shredded return) with increases of 18%–25% over CK, but falling back to control levels under full returns (T3/T4). Conversely, *Thermoleophilia* continued to rise, reaching 32% above CK in T4. Ecologically, the half-rate return (T2) may have provided an optimal C:N ratio that triggered a synergistic bloom of the eutrophic *Gammaproteobacteria* and *Actinobacteria*, whereas carbon surfeit under T3/T4 favoured the heat-tolerant *Thermoleophilia*, whose thermostable enzymes drive late-stage, high-temperature fermentation of residues.

**Figure 10:**
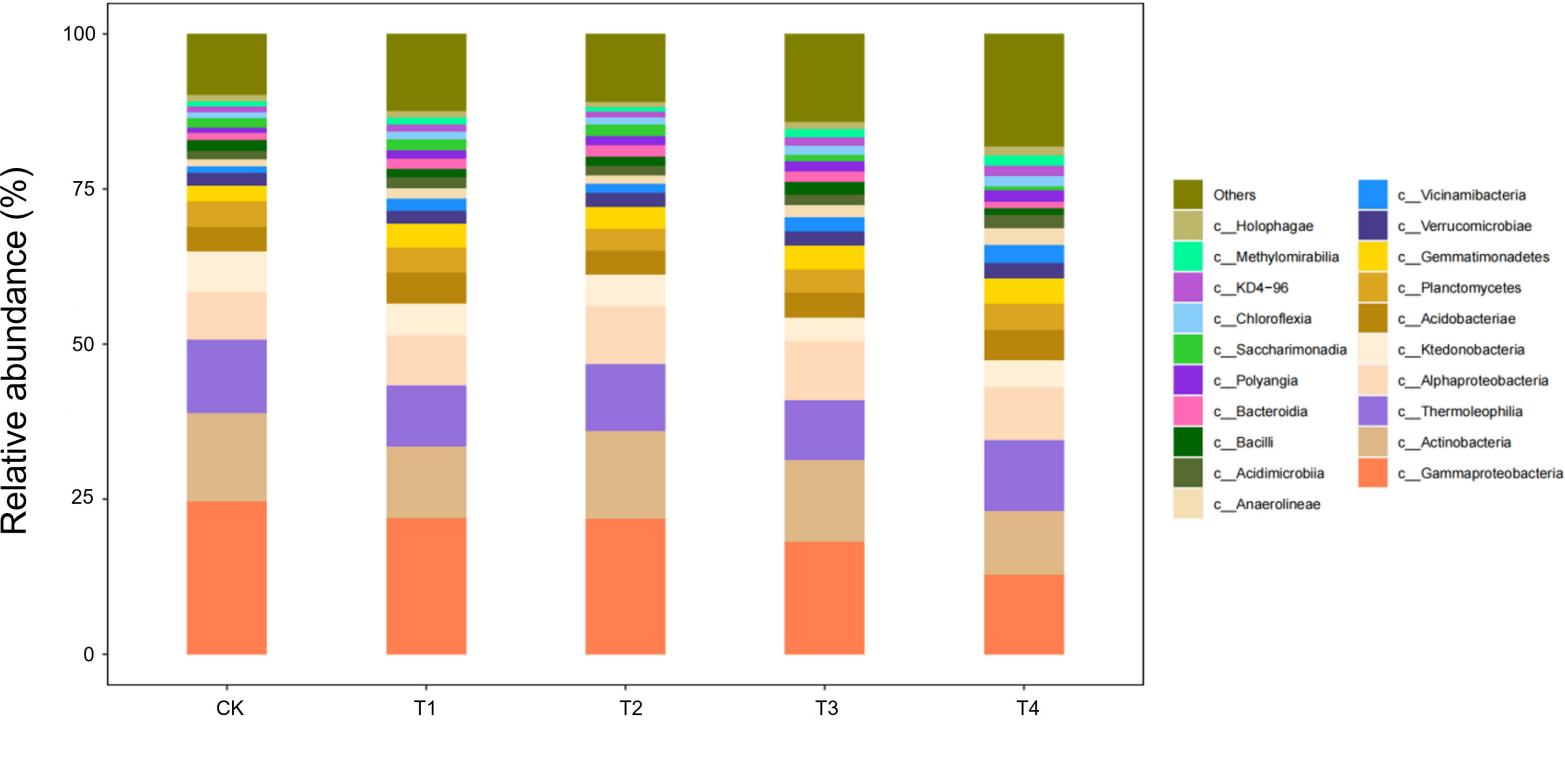
Class-level soil microbial composition at the boll-opening stage is altered by straw incorporation.

Boll-opening-stage communities exposed the terminal effects of straw decomposition (**Figure 10**). Relative to the control, *Gammaproteobacteria* and *Actinobacteria* declined by 15%–18% under every return regime (T1–T4), whereas *Thermoleophilia* followed a unimodal pattern, peaking in T2 with a 28% increase over CK. Ecologically, prolonged carbon depletion combined with the accumulation of phenolic by-products under full returns (T3/T4) curbed the eutrophic taxa (*Gammaproteobacteria*/*Actinobacteria*). By contrast, the thermophilic *Thermoleophilia*, equipped with heat-stable enzymes, exploited lignin-derived end-products most efficiently at the intermediate dose (T2), securing niche dominance. Across the season, T3/T4 initially stimulated eutrophic guilds (+12%–15% *Gammaproteobacteria* at the seedling stage); T2 established a balanced core consortium (+25%) from bud to flower–boll stages; only T2 maintained community stability at boll opening by synchronising C–N release. Thus, a two-thirds shredded return (T2) emerges as the optimal strategy for season-long microbial sustainability.

#### 3.5.3 Number of soil microbial OTUs throughout the entire growth period

**Figure 11:**
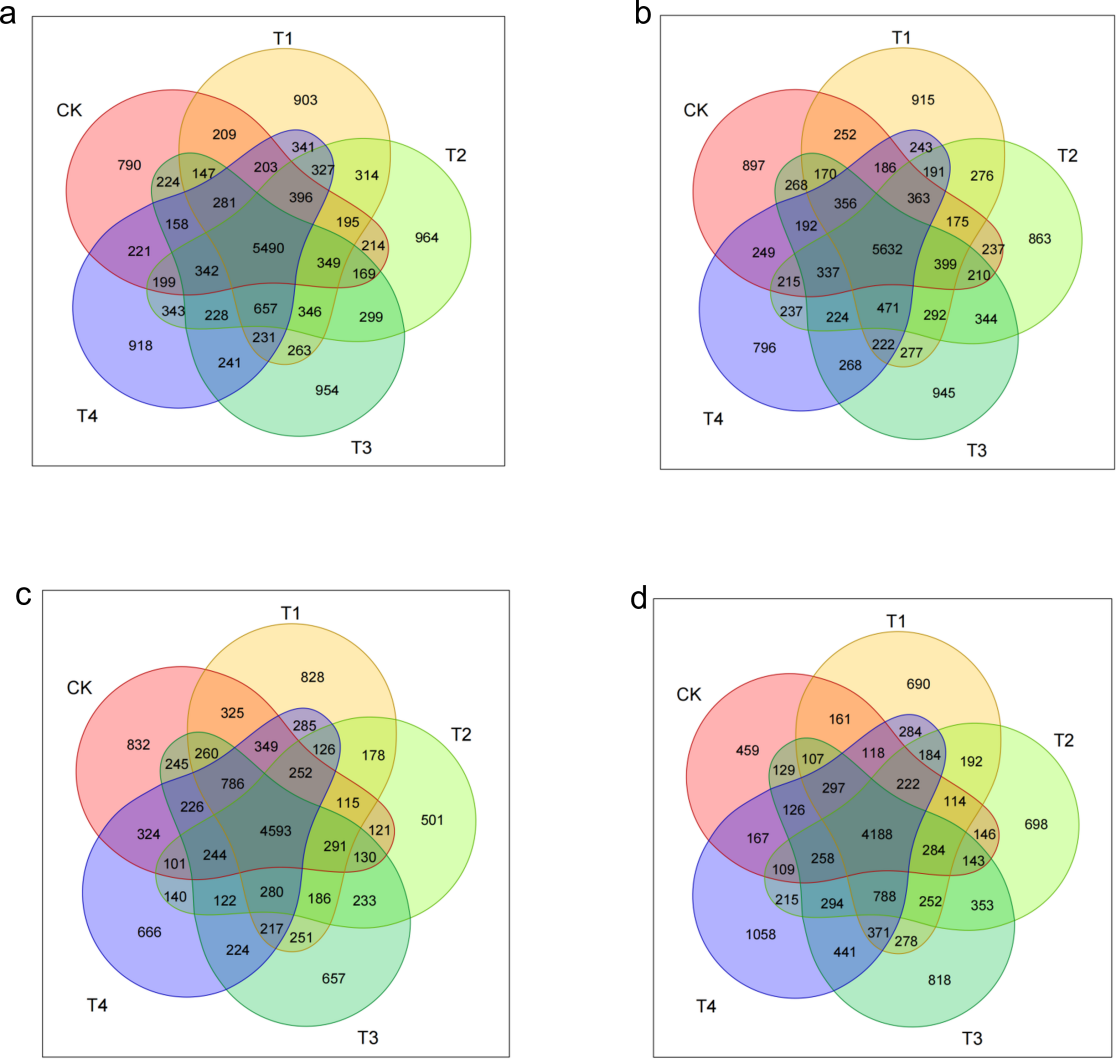
Dynamic shifts in soil microbial richness and community complexity in response to straw incorporation. **(A–D)** Venn diagrams depicting the number of shared and unique operational taxonomic units (OTUs) across different straw management practices at the seedling **(A)**, bud **(B)**, flower–boll **(C)**, and boll-opening **(D)** stages.

As illustrated in **Figure 11**, the seedling-stage soils contained 5,490 shared OTUs across treatments, yet the number of unique OTUs followed a clear gradient: T2 peaked at 964, a 22.0% rise over CK (790); T3 (954), T4 (918), and T1 (903) were 20.8%, 16.2%, and 14.3% higher than CK, respectively (*p* < 0.05). Thus, shredded straw return (T1–T3) increased carbon availability and promoted community differentiation, with the two-thirds rate (T2) producing the greatest gain in structural complexity.

By the bud stage, the shared OTU pool expanded to 5,632, yet treatment effects diverged: unique OTUs under T3 (945) were 5.4% higher than CK (897), whereas natural mulching (T4) dropped to 796, 11.2% below CK. This confirms that shredding (T3) enlarges microbial niches via accelerated straw mineralisation, while surface residue (T4) physically restricts community expansion.

During the flower–boll stage, the shared OTU count dropped to 4,593, and every straw treatment reduced community specificity: T2 unique OTUs plunged to 501, 39.8% below CK (832); T3 (657), T4 (666), and T1 (828) declined by 21.0%, 20.0%, and 0.5%, respectively. The collapse coincides with peak metabolic stress from straw decomposition, with the C:N imbalance under T2 (two-thirds shredding) exerting the strongest suppression.

At the boll-opening stage, the shared OTU base shrank to 4,188, but straw return reversed earlier inhibition: unique OTUs in T4 (1,088) surged 137.0% over CK (459); T3 (818), T2 (698), and T1 (690) rose by 78.2%–50.3%. This highlights the late-season rebound driven by delayed carbon release under T4, while T3 still retains high specificity.

Overall, straw return drove a three-stage trajectory in community complexity. In the early stage (seedling), shredded returns (T1–T3) raised unique OTUs by 14.3%–22.0% over CK, with T2 peaking at 964 (+22.0%), evidencing rapid-carbon stimulation. In the middle stage (bud to flower–boll), natural cover (T4) reduced uniqueness at the bud stage (−11.2%), and all treatments suffered OTU collapse (−0.5%–39.8%) at flowering, T2 most severely (−39.8%), reflecting C–N imbalance. In the late stage (boll opening), T4’s delayed carbon release triggered a 130.5% explosion in unique OTUs, while T3 still posted a 78.2% gain. Thus, T2 delivers “fast response–high risk” (early boost, mid-season crash), whereas T4 offers “delayed response–high resilience,” avoiding mid-season stress and excelling late.

### 3.6 Changes in cotton yield under different treatments

Straw return significantly enhanced cotton productivity by synergistically optimising yield components, with full-rate return (T3/T4) showing the greatest effect (**Table 3**). Boll number per plant increased linearly with straw input (CK 14.87 → T4 20.33); T3 (20.07) and T4 (20.33) were 35.0% and 36.7% higher than CK (*p* < 0.05). Single-boll weight rose by 9.0% and 10.4% under T3 (4.61 g) and T4 (4.67 g) relative to CK (4.23 g), while lint percentage concurrently reached 36.1% in both treatments. This synergy drove yield surges: seed-cotton output under T4 (5,700.16 kg ha^−1^) and T3 (5,557.08 kg ha^−1^) exceeded CK (3,768.44 kg ha^−1^) by 51.3% and 47.5%, and lint yield rose by 63.8% (T4 2,055.63 kg ha^−1^) and 59.8% (T3 2,004.79 kg ha^−1^) over CK (1,254.90 kg ha^−1^). Thus, full-rate straw return—particularly T3—simultaneously boosts boll set, boll size, and lint turnout, achieving dual breakthroughs in seed- and lint-cotton yields (>47%), with mulching (T4) offering a slight edge in boll number and lint yield.

**Table 3:** Effect of different treatments on yield traits and yield of cotton.

Treatment	Boll number per plant/pieces	Boll weight/g	Lint percentage/%	Seed cotton yield/kg·ha^–1^	Lint yield/kg·ha^–1^
CK	14.87 ± 0.99d	4.23 ± 0.19b	33.29 ± 0.77c	3,768.44 ± 189.67c	1,254.90 ± 74.06c
T1	17.07 ± 0.70c	4.26 ± 0.06b	33.88 ± 0.55bc	4,358.40 ± 175.67bc	1,477.11 ± 75.33bc
T2	18.13 ± 1.01bc	4.40 ± 0.20ab	35.44 ± 1.25ab	4,789.76 ± 386.87b	1,699.46 ± 178.46b
T3	20.07 ± 1.03ab	4.61 ± 0.10a	36.10 ± 0.94a	5,557.08 ± 376.31a	2,004.79 ± 108.97a
T4	20.33 ± 1.50a	4.67 ± 0.09a	36.07 ± 1.25a	5,700.16 ± 312.64a	2,055.63 ± 120.69a

Different lowercase letters indicate significant differences in mean values between different treatments during the same growth stage, as determined by Duncan’s one-way ANOVA (*p* < 0.05).

### 3.7 Changes in fibre quality of cotton under different treatments

Straw incorporation exerted only limited improvements on fibre quality. As shown in **Table 4**, fibre strength was the sole parameter to respond significantly, peaking under T4 at 31.20 cN tex^−1^—an increase of 2.6% over CK (30.40 cN tex^−1^, *p* < 0.05). Other key traits—average length of the upper half (30.07–30.16 mm), uniformity index (84.50%–85.30%), micronaire value (4.63–4.87), and elongation rate (6.17%–6.23%)—did not differ amongst treatments (*p* > 0.05). These results indicate that full-rate surface mulching (T4) selectively enhanced fibre-cell wall development, as evidenced by the 2.6% rise in fibre strength, length, uniformity, and fineness that remained unchanged. Thus, the influence of straw return on fibre quality is highly trait-specific.

**Table 4:** Effect of different treatments on fibre quality of cotton.

Treatment	Average length of the upper half/mm	Uniformity index/%	Fibre strength/cN·tex^–1^	Micronaire value	Elongation rate/%
CK	30.07 ± 0.31a	84.77 ± 0.72a	30.40 ± 0.70ab	4.63 ± 0.15a	6.23 ± 0.15a
T1	29.73 ± 0.32a	84.50 ± 0.79a	29.47 ± 1.15b	4.73 ± 0.06a	6.23 ± 0.06a
T2	29.81 ± 0.26a	84.83 ± 0.50a	30.70 ± 0.17ab	4.80 ± 0.10a	6.23 ± 0.25a
T3	30.10 ± 0.26a	84.53 ± 0.84a	30.40 ± 0.78ab	4.73 ± 0.12a	6.20 ± 0.10a
T4	30.16 ± 0.40a	85.30 ± 0.53a	31.20 ± 1.11a	4.87 ± 0.25a	6.17 ± 0.06a

Different lowercase letters indicate significant differences in mean values between different treatments during the same growth stage, as determined by Duncan’s one-way ANOVA (*p* < 0.05).

## 4 Discussion

This study systematically demonstrates that straw return triggers a three-tier regulatory cascade—physiological–biochemical responses, C–N cycling synergy, and microbial community succession—that collectively restructures and optimises the cotton-field ecosystem. The key findings are summarised in the subsections that follow.

### 4.1 Mechanistic regulation of cotton physiological and biochemical traits by straw incorporation

The results show that by refining C–N cycling, straw return markedly enhances both cotton physiological activity and soil fertility across all growth stages. Under the T3 regime (full-rate shredded return), nitrate-reductase activity surpassed that of CK, T1, and T2 at the seedling, bud, flower–boll, and boll-opening stages, peaking at a 74.1% increase—corroborating the observation of Smith et al. that carbon inputs stimulate plant development (Smith and Stitt, 2007). Concomitant increases in soil organic matter (+10.3%) and alkaline-hydrolysable N (+113.0%) validate the “nitrogen-pool expansion” hypothesis of Tian et al (Tian et al., 2019), whereby straw return enlarges total N reserves and accelerates N turnover through priming effects. Regarding the antioxidant system, peroxidase activity at the flower–boll stage increased by 47.7% (T4) and 46.1% (T3) over CK, lending support to the hypothesis of Malik et al. that humic-like substances enhance membrane stability (Malik et al., 2023).

Collectively, these findings indicate a dual-path pattern in physiological responses: a “rapid-response” mode under shredding returns and a “delayed-robust” mode under surface mulching, offering a theoretical basis for stage-specific residue-return strategies. The transition from an early shredding advantage to a late mulching benefit adds a phenological dimension to the understanding of antioxidative regulation.

### 4.2 Reconstruction of soil physicochemical properties under straw incorporation

Straw return rebuilds soil fertility via three synergistic mechanisms: progressive organic-matter accrual, enlargement of the nitrogen reservoir, and establishment of a pH-buffering system. Regarding soil organic matter (SOM) dynamics, T3 and T4 markedly raised organic-matter levels during the bud and flower–boll stages, whereas no differences emerged at the seedling or boll-opening stage. This pattern corroborates the stage-specific decomposition model proposed by Guan et al (Guan et al., 2020). Concerning N-pool expansion and supply synchrony, T3 boosted total N by 17.7% at the flower–boll stage—precisely coinciding with peak crop N demand—thereby extending current knowledge of priming-induced N mobilisation under cotton straw return (Zhou et al., 2024). For pH buffering, T3 raised soil pH by >8% during the bud and flower–boll stages, corroborating earlier reports that cotton straw can neutralise soil acidity (Zhu et al., 2023). The organic acid anions (e.g., oxalate) released from shredded straw chelate Ca²^+^/Mg²^+^, thereby mitigating the abiotic stress of inherent soil acidification on cotton growth (Tuersun et al., 2024).

### 4.3 Ecological rationale for straw-induced microbial succession

Our findings show that straw return re-assembles the soil microbiome by reshaping carbon-source availability. Directed acclimation is evident: at the seedling stage, *Gammaproteobacteria* (labile-C degraders) increased by 15% under T3; *Actinobacteria* (lignin decomposers) peaked under T2 at the bud stage, corroborating the activation of functional guilds reported by Wang et al (Wang et al., 2020). At boll opening, *Thermoleophilia* dominated—rising 32% in T4—highlighting the selective pressure of surface mulching for thermophilic taxa. *Thermoleophilia*, which thrives in high-carbon environments, is particularly known for its ability to degrade complex organic compounds such as lignin and cellulose, playing a crucial role in the decomposition process in straw-amended soils. Studies have shown that members of this group are well-adapted to conditions of high carbon and low nitrogen, where they help mineralise carbon and contribute to soil carbon cycling. Community diversity exhibited a dual-threshold pattern (dose and time): the Shannon index in T2 fell below T3 at flowering, indicating that a two-thirds return rate is a metabolic-stress threshold, whereas the Chao1 index in T4 exceeded CK at boll opening, confirming a time-lagged rebuilding effect of slow-release C (Huang et al., 2012). Thus, straw may transiently suppress diversity but enhances it in the long term, complementing earlier studies (Chang et al., 2021).

### 4.4 Synergistic mechanisms of yield formation under straw return

This study quantitatively defines a “triple pathway” whereby yield components respond in concert. Under T4, boll number per plant, single-boll weight, and lint percentage rose simultaneously by 36.7%, 10.4%, and 36.1%, respectively, driving a 63.8% jump in lint yield. This cross-regional evidence supports the semi-arid findings of Zhang et al. (2016) that yield gains hinge on coordinated optimisation of sink capacity, sink strength, and harvest index. In addition, the full-rate shredded return (T3) in Hunan’s short-season system boosted yield by >47%, far surpassing the 4.2% increase reported by Liao et al. (2018) for rice, underscoring cotton’s exceptional responsiveness to straw incorporation.

### 4.5 Selective regulation of fibre quality by straw return

Our study shows that fibre-quality traits respond unevenly to residue-return modes. Fibre strength was the only parameter to increase significantly, rising by 2.6% under T4 relative to CK. This aligns with the findings of Jin et al (Jin et al., 2024). A plausible mechanism is that humic substances released late in the decomposition of surface-mulched straw promote a more ordered alignment of cellulose microfibrils; confirmation will require microstructural analysis. Other traits—average length of the upper half, micronaire value, etc.—were unaffected, supporting the view that fibre quality is largely genotype-controlled (Naoumkina and Kim, 2023). Future work should explore how residue regimes modulate the expression of key fibre-development genes (e.g., *GhEXP1*). For practice, we recommend T4 mulching in premium textile-cotton zones to gain both higher strength and yield, whereas the more economical T3 shredding regime suits mainstream production.

In sum, by integrating physiological–biochemical adjustments, fertility reconstruction, and microbial succession, straw return systematically enhances cotton-field functionality, underpinning precision agronomic strategies for different regions.

### 4.6 Future perspectives

Straw mulching practices have great potential to enhance both cotton yield and soil health, but their widespread adoption requires robust monitoring and policy support. Integrating soil microbial activity indicators into the existing Soil Health Card framework could provide farmers with a more comprehensive understanding of soil health. By incorporating microbial biomass, diversity, and enzyme activities, the Soil Health Card could not only assess the physical and chemical properties of soil but also track biological factors crucial for sustainable soil management.

For example, full-rate shredded straw return (T4), which promotes microbial diversity, could be monitored through the Soil Health Card to offer farmers clear guidance on the benefits of straw mulching. This would help institutionalise sustainable practices and encourage wider adoption at the farm level.

This approach draws from successful models like India’s Soil Health Card Scheme (Reddy, 2019) and could be adapted to other regions. Incorporating microbial health indicators into policy frameworks would provide farmers with valuable tools for enhancing soil sustainability while contributing to broader goals of sustainable agriculture and climate resilience (Amarender Reddy and Prasad, 2025).

## 5 Conclusions

By simultaneously enhancing plant physiological activity and soil ecological function, straw incorporation markedly improves cotton growth and boosts yield. Full-rate shredded return (T3) dominates the benefits from the seedling to the flower–boll stage, whereas full-rate shredded return (T4)—after complete straw decomposition at the boll-opening stage—triggers an explosion in microbial diversity (higher OTU richness) and selectively raises fibre strength. Overall, full shredded incorporation (T4) emerges as the optimal strategy for balancing high yield with soil health.

## Data Availability

The original contributions presented in the study are included in the article/supplementary material. Further inquiries can be directed to the corresponding author.
